# Caribou and Reindeer Population Cycles Are Driven by Top‐Down and Bottom‐Up Mechanisms Across Space and Time

**DOI:** 10.1002/ece3.71348

**Published:** 2025-05-07

**Authors:** T. J. Clark‐Wolf, Jack St. John, Chandni A. Rajesh, Mark Hebblewhite

**Affiliations:** ^1^ Department of Wildland Resources and Ecology Center Utah State University Logan Utah USA; ^2^ Wildlife Biology Program, Department of Ecosystem and Conservation Sciences, W.A. Franke College of Forestry and Conservation University of Montana Missoula Montana USA; ^3^ Center for Ecosystem Sentinels, Department of Biology University of Washington Seattle WA USA

**Keywords:** biogeographic gradients, density dependence, density independence, management, predation, *Rangifer tarandus*

## Abstract

Anthropogenic change is reshaping the regulation and stability of animal population dynamics across broad biogeographic gradients. For example, abiotic and biotic interactions can cause gradients in population cycle period and amplitude, but this research is mostly constrained to small mammals. Caribou and reindeer ( *Rangifer tarandus* spp.) are threatened by human‐caused change and are known to fluctuate in population over multidecadal scales. But it is unclear how ecological mechanisms drive these cycles and whether these mechanisms are similar to those found in smaller mammals. Here, we carried out a global biogeographic study of *Rangifer* population cycles in response to top‐down and bottom‐up mechanisms. We hypothesized that predation and food resources would interact to affect the amplitude and period of population cycles across the species' range. To test this, we used a two‐pronged approach: (1) we conducted a range‐wide statistical analysis of population data from 43 *Rangifer* herds; and (2) we built tri‐trophic mechanistic population models of predator–*Rangifer*–food interactions. This approach allowed us to merge theoretical and empirical approaches to better understand the drivers of population cycling across space and time. We found statistical evidence for long‐term cyclicity in 19 *Rangifer* populations, and some evidence that decreasing food productivity and winter temperatures may have caused increased period length and amplitude across spatial gradients. Our mechanistic model largely agreed with our empirical results, showing that decreased food resources and increased predation can drive more intense cycles over time. These paired empirical and theoretical results suggest that gradients in *Rangifer* population cycles match ecological mechanisms found in smaller mammals. Moreover, human‐caused shifts in climate, food resources, and predators may shift *Rangifer* population dynamics towards more booms and busts, threatening population persistence. We recommend that dynamic management strategies, in tandem with theoretical and empirical approaches, could be used to better understand and manage population cycles across space and time.

## Graphical abstract

Caribou and reindeer are threatened by anthropogenic change and are known to fluctuate in population size over centuries, but it is unclear what ecological mechanisms drive these population cycles. We used statistical and mechanistic models based on population data from 43 caribou and reindeer herds and found that both top‐down and bottom‐up drivers altered the amplitude and period of these population cycles. These results suggest that anthropogenic shifts in climate, food resources, and predators may shift caribou and reindeer population dynamics to more booms and busts, threatening population persistence.

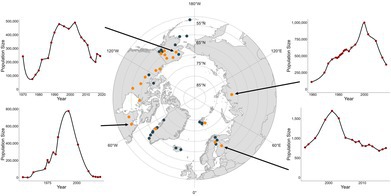

## 1 Introduction

Understanding the stability of animal population dynamics across broad biogeographic gradients is critical, as these complex ecological systems are being reshaped under human‐driven change (McCann 2011; Post 2013). Population cycles have long fueled research focusing on population regulation and stability (Elton 1924), and later, their biogeographic patterns (Kendall et al. 1998). In fact, biogeographic gradients in population cycles are ubiquitous in many animals, arising in, insects, birds, and mammals (e.g., Angelstam et al. 1985; Butler 1951; Hansson and Henttonen 1985; Haynes et al. 2012). However, it is unclear whether and how abiotic and biotic mechanisms produce these gradients in cycle amplitude and period length (Barraquand et al. 2017; Bjørnstad et al. 1995; Turchin 2003). This is increasingly relevant as anthropogenic change can affect abiotic and biotic mechanisms, such as climate change impacts on resources and prey vulnerability to predation.

Population cycles are generally created by delayed (i.e., second‐order) density‐dependent feedback mechanisms, where reproduction or survival of individuals within a population is reduced after reaching high densities (and vice versa for low densities) (May 1972, 1974; Royama 1992; Turchin 1999, 2003). One common top‐down mechanism, the “specialist/generalist predation hypothesis,” suggests that specialist predators are more prominent than generalist predators in northern latitudes, leading to a delayed numerical response of specialist predators to prey, increasing prey population cycle amplitude and period length moving from south to north (Bjørnstad et al. 1995; Hanski et al. 1991; Hanski and Korpimäki 1995). In contrast, bottom‐up mechanisms like the “food hypothesis” posit that the time required for food to regrow after overexploitation can cause populations to cycle, where food resources take longer to regrow in low productivity northern habitats (Ekerholm et al. 2001; Oksanen and Oksanen 1992; Turchin et al. 2000; Turchin and Batzli 2001). In support of both of these hypotheses, experimentally altering both top‐down and bottom‐up factors is known to change the amplitude and period of population cycles (Boutin 1990; Korpimäki and Norrdahl 1998; Krebs et al. 1995; Prevedello et al. 2013). Yet, much of the evidence for the effect of top‐down and bottom‐up drivers on the stability of cycles across biogeographic gradients focuses on short‐generation small mammals in northern Europe (Korpimäki et al. 2004; Oli 2019; Turchin 2003).

Population cycles, and as a whole—complex nonlinear population dynamics—have been thought to be limited to smaller organisms, like insects or rodents, due to their ability to reproduce quickly (Hassell et al. 1976; May 1974; Turchin and Taylor 1992). This is despite long‐term evidence showing complex, nonlinear dynamics including population cycles in large mammals (Caughley 1970; Clutton‐Brock et al. 1997; Forsyth and Caley 2006; Grenfell et al. 1992; Leopold 1943), often driven by spatial gradients in population dynamics (Post 2005; Stenseth et al. 1999). In fact, population cycles have been hypothesized to occur in large mammal species like ungulates (Fryxell et al. 1991; Post et al. 2002; Turchin 2003). Any search for biogeographic patterns in population cycles in large mammals has been hampered by the long‐term data necessary to recognize these cycles, where the period length of a cycle is hypothesized to scale with life‐history characteristics like generation time or body size (Hutchinson 1953; Peterson et al. 1984). In large mammals with generation times > 10 years, it may take decades to collect data to analyze cycles. This is likely what has limited research on the biogeographic extent of population cycles in many large mammal species.

Barrenground caribou and reindeer ( *Rangifer tarandus* spp.; hereafter, “*Rangifer*”) are one large mammal species that has been observed to cycle in abundance over long periods of time, as shown by Indigenous knowledge (Beaulieu 2012; Dokis‐Jansen et al. 2021; Ferguson et al. 1998; Santomauro et al. 2012), population surveys (Bergerud 2008; Gunn 2003; Meldgaard 1986; Messier et al. 1988), and paleoecological evidence (Morneau and Payette 2000; Payette et al. 2003; Zalatan et al. 2006). *Rangifer* are threatened by anthropogenic change, which has been implicated as the main driver of population decline across their range (Fauchald et al. 2017; Festa‐Bianchet et al. 2011; Vors and Boyce 2009). Similar to population cycles in small mammals, top‐down and bottom‐up factors are presumed to drive *Rangifer* population dynamics and consequently their cycles (Festa‐Bianchet et al. 2011; Mallory and Boyce 2018; Vors and Boyce 2009). Predation of *Rangifer* (top‐down) by predators like wolves ( *Canis lupus* ) and brown bears ( *Ursus arctos* ) can be an important regulatory factor affecting population dynamics (Ballard et al. 1997; Bergerud and Elliot 1986; Dale et al. 1994; Gasaway et al. 1983; Klaczek et al. 2016; Seip 1991). Yet in some cases, predation has a minor effect on *Rangifer* populations, as shown by long‐term predator control experiments and other studies (Boertje et al. 2017; Clark and Hebblewhite 2020; Messier 1995; National Research Council 1997; Van Ballenberghe 1985). Bottom‐up factors such as the availability of forage, including lichen, graminoids, and vascular plants (Denryter et al. 2017; Webber et al. 2022) are also important factors that regulate population dynamics (Fauchald et al. 2017; Heggberget et al. 2002; Joly et al. 2007; Klein 1982; Messier et al. 1988). There is also evidence of strong interactive feedbacks between herbivory by *Rangifer* and lichen regrowth (Bernes et al. 2015; Collins et al. 2011; Gough et al. 2008; Hansen et al. 2007; van der Wal et al. 2001) due to the long regrowth period for lichen following over‐exploitation (roughly 40–60 years). Climate change likely interacts with both these top‐down and bottom‐up factors to affect *Rangifer* population cycles (Aanes et al. 2002; Gunn 2003; Joly et al. 2011; Klein 1968; Mallory and Boyce 2018; Tyler 2010). For example, increased winter precipitation may directly reduce forage availability (Collins and Smith 1991) or increase vulnerability to predation (Hegel et al. 2010b; Post and Stenseth 1998; Telfer and Kelsall 1984). What remains an open question is as follows: How do these top‐down and bottom‐up factors interact to modulate *Rangifer* population cycles across broad biogeographic gradients? Moreover, what can we learn about the future of *Rangifer* population cycles and stability under future human‐caused change?

Here we conducted a broad biogeographic analysis of *Rangifer* population cycles in response to top‐down and bottom‐up factors. We hypothesized that, similar to small mammals, both the predation and food hypotheses will affect the amplitude and period of *Rangifer* population cycles (Gunn 2003). Specifically, we hypothesized that stronger predation and limited food availability would drive both longer periods and higher amplitudes. We evaluated these hypotheses through a two‐pronged approach to understand drivers of *Rangifer* population cycles across space and time. First, we collected data on *Rangifer* populations and conducted a statistical analysis to determine the relationship between top‐down and bottom‐up factors and population cycles across their global range. Second, we built tri‐trophic mechanistic population models of predator–*Rangifer*–food interactions, parameterized by previous empirical research, to determine the importance of top‐down and bottom‐up factors over long timescales. In summary, we found some evidence that the stability of *Rangifer* population cycles is driven by top‐down and bottom‐up factors across both time and space.

## 2 Materials and Methods

### 2.1 Data Collection

We collected population estimates for *Rangifer* from scientific papers and management reports. In some cases, management reports updated older population estimates based on new statistical techniques, so we used the most recent version of a management report for population estimates. Initially, we collected estimates of measurement error associated with the population estimates to account for differences in survey methodology. However, many estimates in management reports did not report measurement error, so we instead opted to use statistical tools to characterize population cycles that are robust to observation error (see “Data analysis and model selection”). *Rangifer* population surveys were often not conducted every year for most populations. The mean percentage of time‐series data that were missing was 39.2% (SD = 32.3%). As a result, we imputed the missing data using “stine” interpolation (Stineman 1980) in the *imputeTS* R package (Moritz and Bartz‐Beielstein 2017). Stine interpolation was chosen as it works well for datasets with abrupt changes in slope (Perillo and Piccolo 1991) such as cycles, and we visually assessed interpolated population estimates to ensure fit and that no new inflection points were created. We also conducted a cross‐validation analysis to determine whether imputed data were accurate. We removed 20% of the time‐series data randomly, and then estimated these using imputation. We found that the correlation between the real and imputed time‐series data was 0.958 (SD = 0.07), indicating that the interpolation method creates accurate time‐series data.

We hypothesized that both top‐down and bottom‐up factors would affect *Rangifer* population cycles over space (Gunn 2003). We hypothesized that predators, including wolves, would impact population cycles due to the impacts of predation on small mammal population cycles as well as the effects of wolves on moose population cycles at Isle Royale National Park (Barraquand et al. 2017; Gunn 2003; Post et al. 2002). For most *Rangifer* herd ranges, population estimates of predators did not exist, so we estimated the number of predator species and the presence/absence of wolves as a proxy for predation using current species range maps (Mech and Peterson 2003). We calculated a proxy for primary productivity using dynamic habitat indices (DHIs) from NDVI (Hobi et al. 2017). NDVI has been shown to be a reasonable proxy for vegetation productivity on *Rangifer* ranges year round in the Arctic (Johnson et al. 2018). DHIs encompass the phenological productivity over each year and have been used to measure the dynamics of growing season vegetation productivity that are useful for predicting population dynamics of different species (Hobi et al. 2017). We also hypothesized that there would be an effect of latitude on population cycles due to evidence of large‐scale spatial gradients in *Rangifer* population dynamics (Post 2005). We also collected categorical information from management reports on the biome of the *Rangifer* herd, defined as whether the summer and winter range were in either taiga (boreal forest) or tundra (i.e., “taiga/taiga,” “taiga/tundra,” and “tundra/tundra”). We collected additional weather and habitat data, using a buffer of 10 km around the centroid of the herd location. Average winter monthly minimum temperature and average winter monthly precipitation were collected from TerraClimate (Abatzoglou et al. 2018).

We also collected confounding biological and ecological data that may explain the presence of *Rangifer* cycles among herds. We categorized *Rangifer* herds according to subspecies, such as *Rangifer tarandus pearyi* (Peary caribou), as well as ecotype classification, such as insular or montane, following Mallory and Hillis (1998). These data are likely to explain genetic, ecological, and behavioral differences between *Rangifer* herds (Ahrestani et al. 2013). Lastly, we categorized the herd as either wild or semi‐domesticated, as a few herds were free‐roaming but closely managed.

### 2.2 Data Analysis and Model Selection

Analyses of population cycles, especially in small mammals, typically rely on multiple population fluctuations over many years to draw inference, and then employ methods such as spectral or wavelet analyses to study these cycles (Barraquand et al. 2017). However, *Rangifer* population cycles may be too long to collect enough observational data for multiple cycles, as shown by previous paleoecological and Indigenous evidence of multiple cycles over long periods of time (Gunn 2003). Therefore, we employed a space‐for‐time substitution (Lovell et al. 2023), exploring whether repeated patterns of population cycles across space are indicative of evidence for population cycles in *Rangifer*. Therefore, we statistically analyzed whether *Rangifer* populations were cyclic by using periodogram analysis of each time series using the *peacots* R package (Louca and Doebeli 2015). Herds were considered cyclic if their estimated period was statistically different (*p* < 0.05) when compared against an Ornstein‐Uhlenbeck state‐space null model, which adds temporal correlations to white noise (Louca and Doebeli 2015). In this model, the statistical significance of an estimated period is evaluated against the null hypothesis that some noncyclic process caused the underlying dynamics. In this case, our null hypothesis was based on white noise with added temporal correlations to ensure an accurate description of stochastic processes. These methods have been found to be robust to measurement error, especially to time series without missing values. We did not find a significant effect of time series length on the likelihood of finding a statistically significant cycle (*p* = 0.259). For the herds that were found to be statistically cyclic, we estimated both period and amplitude. Period, the time (in years) of a full population cycle, was calculated as the inverse of the optimized frequency of the fit periodogram. Amplitude was calculated using the following equation: 0.5 × (maximum estimate – minimum estimate)/mean estimate, where amplitude was standardized by the mean and multiplied by 0.5 to give an estimate of the population size between the mean of the wave to its peak or trough. We did not find a significant effect of % imputed data on cycle period (*p* = 0.667) or amplitude (*p* = 0.102).

We then used generalized linear models (GLMs) with Gaussian errors and an identity link function to explain the relationship between the predictor variables and cycle period and amplitude. We simplified models by backwards selection using a step‐wise procedure based on AIC, repeating until no further reduction in AIC was possible (Burnham and Anderson 2003; Tredennick et al. 2021; Zuur et al. 2009). We removed predictor variables for semi‐domesticity and ecotype due to high collinearity (|*r* > 0.7|) with other predictors. We confirmed model assumptions of normality and homoscedasticity of residuals by examining normal quantile–quantile plots and residuals versus fitted values, respectively.

### 2.3 Tri‐Trophic *Rangifer* Model

In our dual empirical and theoretical approach, we modeled *Rangifer* as part of a vegetation–*Rangifer*–predator model to understand the influence of top‐down and bottom‐up effects on *Rangifer* dynamics over time. These models are based on Rosenzweig–MacArthur predator–prey models (Rosenzweig and MacArthur 1963), which were adapted by Turchin (2003) to model ungulate population dynamics. Consumer–resource dynamics were modeled using hyperbolic Michaelis–Menten functions (i.e., a variant of Holling's disc equation; Real 1977). The tri‐trophic model is described as:

$$\frac{\mathrm{dV}}{\mathrm{dt}}=u_{0}\left(1-\frac{V}{m}\right)-\frac{\mathrm{aVN}}{b+V}$$

$$\frac{\mathrm{dN}}{\mathrm{dt}}=\mathrm{\xi N}\left(\frac{\mathrm{aV}}{b+V}-\eta \right)-\frac{\mathrm{cNP}}{d+N}$$

$$\frac{\mathrm{dP}}{\mathrm{dt}}=\mathrm{ΧP}\left(\frac{\mathrm{cN}}{d+N}-\mu \right)-\frac{s_{0}}{\kappa }P^{2}$$

where *V*, *N*, and *P* represent the vegetation biomass, *Rangifer* densities and predator densities, respectively. Vegetation biomass dynamics were modeled using a regrowth equation instead of a logistic function, as *Rangifer* do not cause high vegetation mortality (Turchin 2003). *u*_*0*_ is the vegetation regrowth rate at *V* = 0, *m* is the maximum vegetation coverage, and *a* and *b* are the *Rangifer* foraging rate and handling time of vegetation as a Type II functional response. In the *Rangifer* submodel, *ξ* is the *Rangifer* conversion efficiency, *η* is the *Rangifer* zero population growth consumption rate (which is used instead of death rate as it is easier to parameterize; Turchin (2003)), and *c* and *d* are the predator foraging rate and handling time of *Rangifer* as a Type II functional response. Lastly, predator dynamics were modeled in part with the Bazykin model, which allows for density‐dependent self‐limitation of predators and is more dynamically stable than the traditional Rosenzweig–MacArthur model (Turchin 2003). *Χ* is the predator conversion efficiency, *μ* is the predator zero population growth (ZPG) consumption rate, *s*_*0*_ is the intrinsic rate of predator increase, and *κ* is the maximum density of predators.

### 2.4 Model Parameterization

The tri‐trophic *Rangifer* model was parameterized as follows, with units of vegetation in Mg (dry weight), area in km^2^, time in years, and density of *Rangifer* and predators as individuals/km^2^ (Table 1). Maximum vegetation coverage, *m*, was set to 100 Mg/km^2^, representing previous estimates of the maximum carrying capacity of vascular plants and lichens (Weclaw and Hudson 2004). *Rangifer* foraging rate of vegetation, *a*, was estimated as 2.5 Mg/individual/year as the maximum reported intake rate of vegetation (Holleman et al. 1979; Klein 1982; Trudell and White 1981). We estimated *b*, the *Rangifer* handling time of vegetation, as 25.4 Mg/individual/year, by fitting a nonlinear functional response model to Trudell and White (1981), which shows the relationship between total vegetation biomass and *Rangifer* food intake. We set the *Rangifer* ZPG consumption rate, *η*, to be 0.89 Mg/individual/year, which Turchin (2003) estimated to be half of the average intake rate of vegetation (Klein 1982). *Rangifer* conversion efficiency of vegetation, *ξ*, was calculated following Turchin (2003), where:

$$\xi =\frac{r_{0}}{\frac{\mathrm{am}}{\left(b+m\right)}-\eta }$$

**TABLE 1:** Parameter values in our tri‐trophic mechanistic model of predator–*Rangifer*–vegetation dynamics.

Parameter	Meaning	Value(s)	Source(s)
u_0_	Vegetation regrowth rate	0.8	This study; Yarranton 1975; Gaare 1997
m	Maximum vegetation coverage	100	Weclaw and Hudson 2004
a	*Rangifer* foraging rate of vegetation	2.5	Holleman et al. 1979; Klein 1982
b	*Rangifer* handling time of vegetation	25.4	This study; Trudell and White 1981
ξ	*Rangifer* conversion efficiency of vegetation	0.27	This study; Heard 1990
η	*Rangifer* ZPG consumption rate	0.89	This study; Turchin 2003
c	Predator foraging rate of *Rangifer*	18.5	Holleman and Stephenson 1981; Dale et al. 1994; Hayes et al. 2000
d	Predator handling time of *Rangifer*	0.5	Dale et al. 1994
Χ	Predator conversion efficiency of *Rangifer*	0.114	Serrouya et al. 2020
μ	Predator ZPG consumption rate	9.25	Mech and Peterson 2003
s_0_	Intrinsic rate of predator increase	0.3	Turchin 2003
κ	Maximum predator density	0.1	Turchin 2003

Abbreviation: ZPG, Zero population growth.

Using *r*_*0*_ (intrinsic rate of *Rangifer* population growth) of 0.3 (Heard 1990), we estimated *ξ* to be 0.27. We estimated *u*_*0*_, the regrowth rate of vegetation, to be 0.8 Mg/km/yr, based on previous estimates of the annual maximum regrowth of *Rangifer* forage (Weclaw and Hudson 2004).

The predator foraging rate of *Rangifer*, *c*, was set to 18.5 *Rangifer*/predator/year based on averaging wolf functional responses on *Rangifer* (Dale et al. 1994; Hayes et al. 2000; Holleman and Stephenson 1981). We roughly estimated *d* to be approximately 0.5 *Rangifer*/km^2^ based on the approximate half‐saturation point of a prior wolf–*Rangifer* functional response (Dale et al. 1994). The predator conversion efficiency of *Rangifer*, *Χ*, was estimated as 0.114 (Serrouya et al. 2020) and *μ*, the predator ZPG consumption rate, was set to be 9.25 wolves/km^2^ based on past estimates of wolf densities (Mech and Peterson 2003). Lastly, *s*_*0*_, the intrinsic rate of predator increase, and *κ*, the maximum density of predators, were estimated to be 0.3 and 0.1, respectively, based on past estimates for wolves (Turchin 2003).

We simulated the tri‐trophic model for 3000 years, discarding the first 1500 years in order to remove transient dynamics, and set the initial conditions for *V*, *N*, and *P* to be 50, 2, and 0.001 individuals/km^2^. Ordinary differential equations were solved using the *ode45* integrator (Dormand and Prince 1980) in the *deSolve* R package (Soetaert et al. 2010). The period and amplitude of the simulated *Rangifer* cycles (*N*) were calculated as previously with empirical *Rangifer* data in “Data analysis and model selection” in order to compare with empirical estimates. In addition, we note that we calculated a discrete, stochastic version of the tri‐trophic model by adding in environmental stochasticity using the *nimble* R package (de Valpine et al. 2023), which produced similar results to the deterministic, continuous model.

### 2.5 Sensitivity Analysis

To compare these simulated results with our statistical analysis of empirical *Rangifer* data, we varied both top‐down (*c*, *d*) and bottom‐up (*u*_*0*_, *m*) parameters that affected the period and amplitude of *Rangifer* cycles. To assess bottom‐up effects, we ran simulations with our tri‐trophic model and simultaneously varied *u*_*0*_ (vegetation regrowth rate) and *m* (maximum vegetation coverage) from 0.1 to 2 and 10 to 200, respectively. To assess top‐down effects, we simultaneously varied *c* (predator foraging rate) and *d* (predator handling time) from 0.01 to 40 and 0.01 to 1, respectively. For all sensitivity analyses, we calculated the period and amplitude of *Rangifer* cycles as done previously. To determine the importance of parameter values to our cycle period and amplitude, we also conducted a wider sensitivity analysis by varying all parameters by −30%, −15%, 0%, +15%, and +30% and measuring period and amplitude.

## 3 Results

**FIGURE 1:**
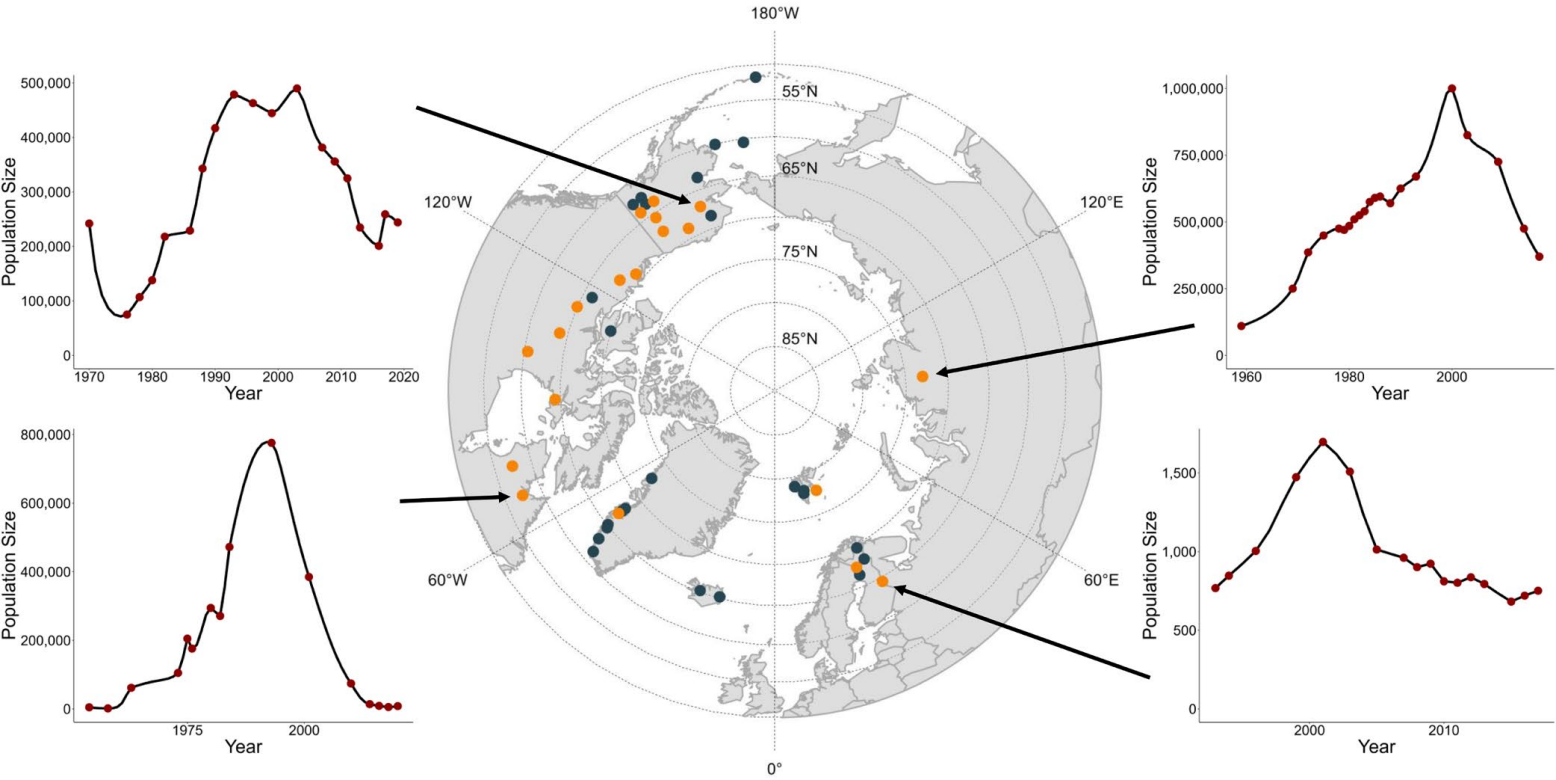
Geographic location of 42 *Rangifer* (caribou and reindeer) populations used in our statistical analysis. Orange dots show the 19 *Rangifer* herds which were statistically found to be cyclic. Population trends are shown for (clockwise, starting at top‐left): Western Arctic, Alaska, USA; Taimyr, Russia; Kainuu, Finland; and George River, Quebec, Canada. Red dots show observed population values, and black lines show interpolated trends.

**FIGURE 2:**
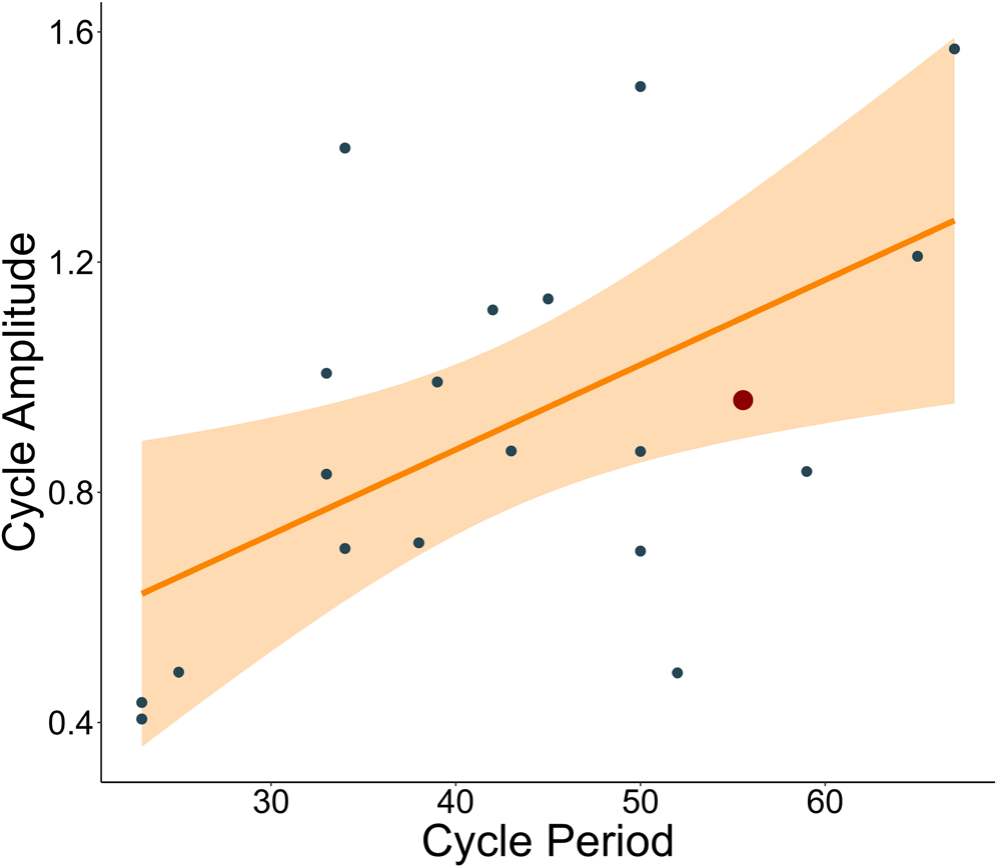
Relationship between *Rangifer* (caribou and reindeer) cycle period and amplitude in our statistical model. Blue dots represent empirical *Rangifer* data, whereas the red dot represents the theoretical period and amplitude of *Rangifer* population cycles from our tri‐trophic model. Orange line and shading indicate the mean and 95% CI of a linear model fit to the trend of this relationship. See an example of the predicted cycle in Figure S1.

We collected long‐term empirical data for 43 *Rangifer* herds, spanning a longitudinal gradient of ~270° across three continents (Figure 1; Table S1). The imputed time‐series length averaged 45.1 years (95% CI: 23–74 years). We found that 19 of the 43 *Rangifer* herds had statistical evidence of population cycles (Figure 2). We estimated that the average period length of *Rangifer* cycles was 42 years (95% CI: 23.0–66.1 years), and the average amplitude was 0.91 (95% CI: 0.418–1.54).

**FIGURE 3:**
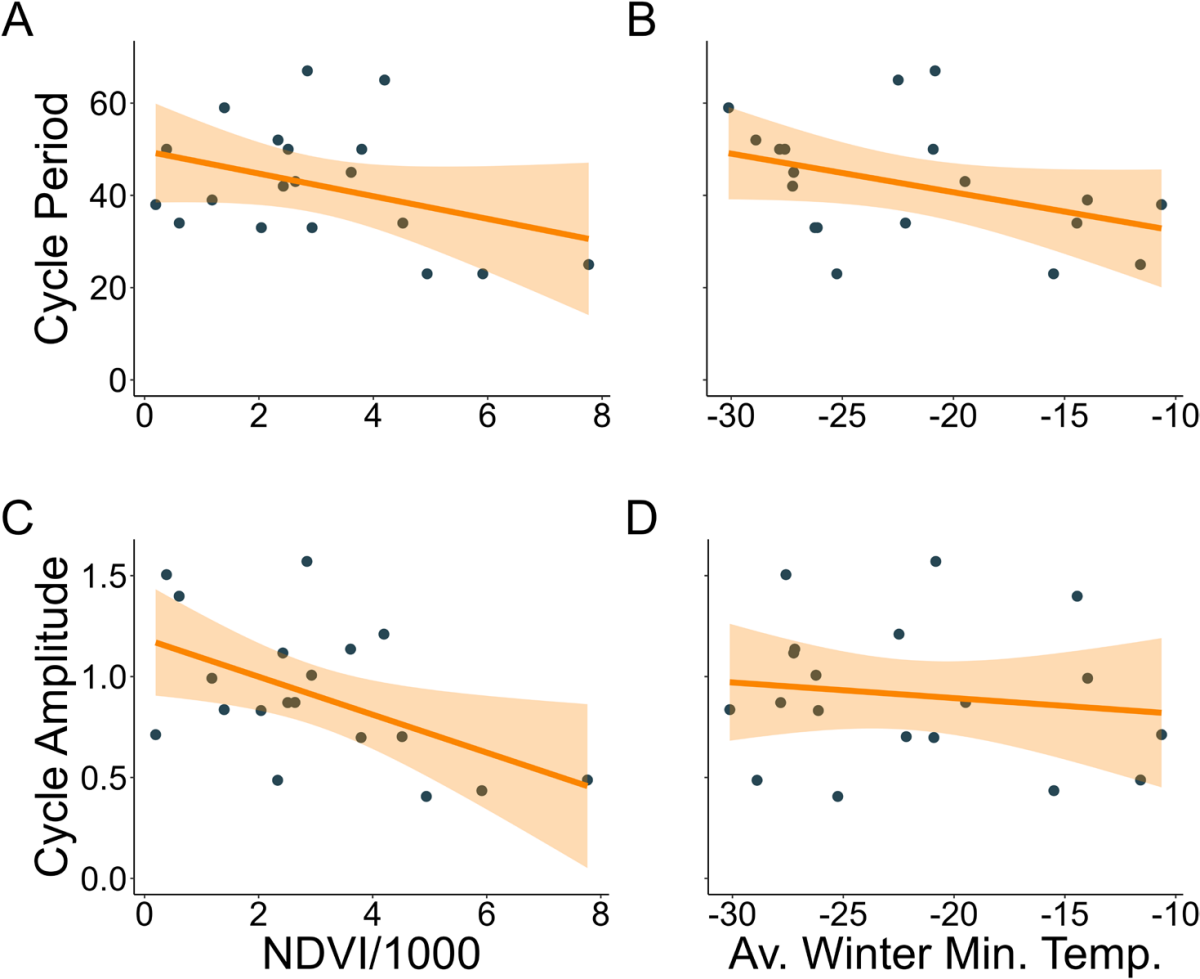
Relationship between NDVI and average winter minimum temperatures and *Rangifer* (caribou and reindeer) cycle period and amplitude in our statistical model. Blue dots represent empirical *Rangifer* data. Orange lines and shading indicate the means and 95% CI of a linear model fit to the trend of this relationship.

We found that bottom‐up effects influenced both *Rangifer* cycle period and amplitude, yet these relationships held significant variability (Tables S2–S5). Both NDVI and average winter minimum temperature were somewhat related to decreasing *Rangifer* cycle period (*β* = −1.596, SE = 1.515 and *β* = −5.568, SE = 2.973, respectively) (Figure 3A,B) and amplitude (*β* = −0.094, SE = 0.037 and *β* = −0.007, SE = 0.013, respectively) (Figure 3C,D). We also observed a moderate negative relationship between latitude and *Rangifer* cycle period (*β* = −9.481, SE = 5.894). In addition, there was a strong positive relationship between cycle period and amplitude (*β* = 7.203, SE = 2.582). However, we did not find any relationship between top‐down effects (e.g., presence/absence of wolves; number of predator species) and *Rangifer* cycle period and amplitude. Lastly, we found that *Rangifer* biology, like subspecies and biome, had some influence on population cycles (see Tables S3 and S5).

**FIGURE 4:**
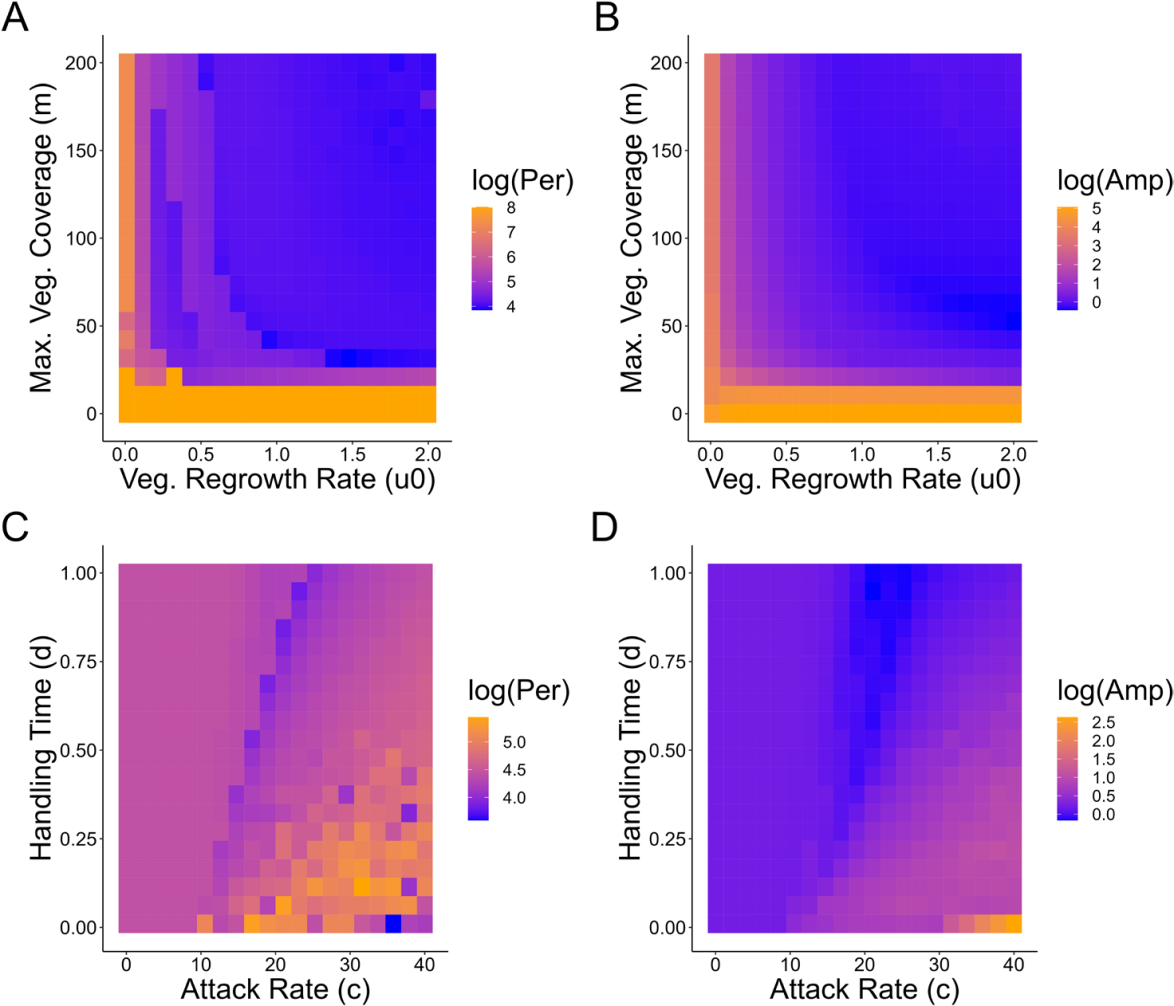
Relationship between bottom‐up (vegetation regrowth rate; maximum vegetation coverage) and top‐down (attack rate; handling time) factors on *Rangifer* (caribou and reindeer) population cycle period and amplitude in our tri‐trophic mechanistic model. Period and amplitude were log transformed for better visualization.

Our tri‐trophic *Rangifer* mechanistic model produced cyclic population dynamics within the range of variation found within our empirical *Rangifer* data (period = 57.9; amplitude = 0.95; Figure 2). Similar to our statistical analysis of empirical *Rangifer* data, we found that bottom‐up factors (vegetation regrowth rate and maximum vegetation coverage) had a negative effect on both *Rangifer* cycle period and amplitude (Figure 4A,B), indicating that increasing vegetation productivity decreased both *Rangifer* cycle period and amplitude. Top‐down factors also influenced *Rangifer* population cycles in our tri‐trophic model. Increasing predator attack rates on *Rangifer* increased both cycle period and amplitude, whereas increasing predator handling times of *Rangifer* decreased cycle period and amplitude (Figure 4C,D). We found evidence of cycles across our wider sensitivity analysis with period and amplitude falling within the range of our empirical *Rangifer* data (Tables S6 and S7).

## 4 Discussion

We found some evidence that large mammal population cycles can display biogeographic gradients. This pattern matches results from rodents and other small mammals in northern Europe and North America (Bjørnstad et al. 1995; Hansson and Henttonen 1985; Kendall et al. 1998). The convergence in our results across statistical and mechanistic models indicates that the ecological mechanisms driving large mammal cycles are similar to those driving small mammal cycles, but over much longer timescales (Erb et al. 2001; Gunn 2003; Peterson et al. 1984). Using a two‐pronged approach of statistical and mechanistic analyses, we found some evidence that *Rangifer* population cycles are sensitive to both top‐down and bottom‐up mechanisms across space and time. This is particularly striking as our tri‐trophic mechanistic model estimated *Rangifer* population cycle amplitude and period within the estimates from our statistical analysis of long‐term *Rangifer* population data (Figure 2). Differences between our statistical analysis of population data and our mechanistic model are likely related to observation error and length of population monitoring. Our research indicates that the stability, persistence, and biogeographic gradients of population cycles in *Rangifer* and other large mammals can be threatened as humans reshape ecosystems, including climate, resources, and predators (Fauchald et al. 2017; Gunn 2003; Mallory and Boyce 2018; Post 2013). Future research could explore how these top‐down and bottom‐up mechanisms correlate with population growth and decline in this species and how these mechanisms vary across space and time.

Our results reinforce the importance of consumer–resource dynamics driving population cycles and stability (McCann 2011). Both top‐down and bottom‐up mechanisms (in support of both the specialist/generalist predation and food hypotheses) caused gradients in *Rangifer* population cycle period and amplitude, matching past research in small mammals (Hanski et al. 1991; Oli 2019; Turchin and Batzli 2001). Predation of *Rangifer* was an important regulatory factor (Bergerud and Elliot 1986; Dale et al. 1994; Gasaway et al. 1983) as higher predation rates caused increased cycle instability, similar to theoretical expectations and empirical results from specialist predators (Hanski et al. 1991; McCann 2011; Murdoch et al. 2002; Turchin 2003). *Rangifer* populations at locations with fewer resources (i.e., lower NDVI) may have higher density‐dependent intraspecific competition over limited forage (Cuyler 2007; Ehlers 2022; Gunn et al. 2003). More specifically, we hypothesize that lower resource productivity may lead to more intraspecific competition, increasing population booms and busts. Density‐dependent top‐down and bottom‐up factors likely interacted with density‐independent climatic features, leading to reduced forage availability and increased predation rates in worse winters (Joly et al. 2011). Our research could be extended by linking climatic features with advanced forms of functional responses in our mechanistic model (e.g., impact of snow on wolf hunting success and delayed density‐dependent predation on *Rangifer*). Our results indicated that *Rangifer* in habitats with more severe weather events had population cycles with longer periods and higher amplitudes, supporting previous research on the effects of colder, severe winters on *Rangifer* populations (Aanes et al. 2002; Gates et al. 1986; Klein 1968; Miller and Gunn 2003; Tews et al. 2013; Tyler 2010). One important extension of this research could be to investigate the influence of large‐scale climate patterns (i.e., teleconnections), like the North Atlantic, Pacific Decadal, and Arctic Oscillations, on *Rangifer* population cycle gradients, which have been studied in regional *Rangifer* populations (Aanes et al. 2002; Couturier et al. 2009; Gunn 2003; Hegel et al. 2010a; Joly et al. 2011; Post and Stenseth 1999). Future research could also explore the Moran effect, where *Rangifer* population cycles may synchronize over space in response to teleconnections (Hansen et al. 2020).

The period length of *Rangifer* population cycles necessitates very long‐term monitoring data, which may have limited our statistical model and results. Typical analyses of population cycles, especially in small mammals (Barraquand et al. 2017), allow for the use of advanced spectral or wavelet analyses to understand cycling over multiple fluctuations. Due to limitations on *Rangifer* monitoring data, we instead used a space‐for‐time substitution, inferring that repeated *Rangifer* population cycles across space robustly indicate the presence of population cycles in this species. Despite this, our statistical analysis was limited by population monitoring, as population data could have represented one full population cycle or less, and estimated period length was limited by the length of the data (Table S1), indicating that further long‐term monitoring efforts of *Rangifer* are necessary to understand population cycles. There are other potential limitations to these population monitoring data. First, many *Rangifer* population estimates in management reports and publications did not provide an estimate of error associated with the observation process. We accounted for this by using periodogram analysis that is robust to observational error by using state‐space models incorporating white noise and temporal autocorrelations (Louca and Doebeli 2015). But these methods, in addition to imputation used to account for missing data, may have introduced additional error into our results. However, prior analysis of *Rangifer* and other ungulate populations indicates that process error far exceeds observation error (Ahrestani et al. 2013), indicating that these data limitations are unlikely to bias our results. Second, although our top statistical model retained bottom‐up predictors like NDVI and minimum temperature, there was significant variation associated with these covariate estimates (Tables S3 and S5), potentially due to error and time‐series length associated with the empirical data. In addition, our top model did not retain any predictors associated with top‐down factors, but this is likely because of limited population data on wolves and other predators in these regions. However, our tri‐trophic mechanistic model (Turchin 2003), parameterized by empirical research, confirmed the importance of these top‐down and bottom‐up factors in our statistical models, despite associated empirical variation. Lastly, the lack of very long‐term monitoring data and shifting cyclic properties of some populations may have limited our ability to find statistically significant cyclic populations. In fact, the majority of the populations analyzed did not have evidence of cyclic properties (Table S1). For example, the Nelchina caribou herd did not have enough statistical evidence to be considered cyclic, despite apparent cycles in data collected from approximately the 1960s–1990s, in part because cycles have dampened since the 1990s (Eberhardt and Pitcher 1992; Van Ballenberghe 1985). We believe that noncyclic dynamics can be attributed to a combination of nonexclusive factors, some of which include: a lack of long‐term monitoring data, shifting cyclic properties due to environmental change and management actions, and ecological drivers of noncyclic dynamics. Future research could investigate those populations that showed no evidence of cyclic attributes and the ecological factors that drive their population dynamics (Post 2005). Longer‐term monitoring of *Rangifer* populations in concert with more advanced statistical techniques (Barraquand et al. 2017) will allow for more robust analysis of *Rangifer* population cycles.

Population cycles can amplify or disappear in response to human‐driven change (Barraquand et al. 2017; Cornulier et al. 2013; Hudson et al. 1998; Ims et al. 2008; Krebs et al. 1995). For example, some population cycles in voles, grouse, and insects have been dampening in period and amplitude in part due to climatic change (Cornulier et al. 2013; Ims et al. 2008). Similarly, the anthropogenic effects of climate change, predator expansion, and management on *Rangifer* populations could lead to population declines (Fauchald et al. 2017; Festa‐Bianchet et al. 2011; Mallory and Boyce 2018; Vors and Boyce 2009), and following our results, changes to population cycles. First, the warming of the Arctic is causing widespread changes in vegetation biomass and productivity (Epstein et al. 2012; Goetz et al. 2005; Ju and Masek 2016), including the expansion of woody shrubs like birch or alder (*Betula nana exilis*, *Betula glandulosa* , and *Alnus viridus*) that are of low forage quality for large herbivores (Christie et al. 2015) and are replacing lichen (Fraser et al. 2014; Macander et al. 2020; Myers‐Smith et al. 2011), an important forage for *Rangifer* during the winter (Denryter et al. 2017; Webber et al. 2022). Our results (Figures 3, 4) indicate that the displacement and reduction of forage in the Arctic could cause there to be more extreme cycles for *Rangifer*, increasing period length and amplitude. In addition, in Alberta and British Columbia, Canada, human‐caused habitat change has led to expanding wolf populations supported by increasing primary prey like white‐tailed deer ( *Odocoileus virginianus* ) or moose ( *Alces alces* ) in the southern fringes of the Arctic, which has been implicated in woodland caribou (*R. t. caribou*) population declines (Latham et al. 2011; Serrouya et al. 2015; Wittmer et al. 2005). Subsequently, increasing predation rates on *Rangifer* via these mechanisms could amplify population cycles. We found a strong correlation between cycle period and amplitude, which, coupled with human‐caused trends in top‐down and bottom‐up factors, suggests that cycles could amplify in the future, threatening *Rangifer* population persistence, as phases of very low population densities could lead to local population extinction.

Management strategies of *Rangifer* population cycles could focus on studying the efficacy of top‐down or bottom‐up control, which in some cases have been experimentally applied to control other animal cycles (Bell et al. 2012; Hudson et al. 1998; Korpimäki and Norrdahl 1998). For example, Korpimäki and Norrdahl (1998) excluded vertebrate predators of voles from experimental plots and found that vole population cycles stabilized. Another potential avenue for the management of population cycles is dynamical control theory, which involves adding or removing individuals during certain regions in the population cycle to dampen or eliminate cycling (Desharnais et al. 2001; Hilker and Westerhoff 2007; Tung et al. 2014). For example, recent experiments with *Drosophila* have shown that these strategies can stabilize populations (Sah et al. 2013; Tung et al. 2016a, 2016b). Concurrently, there is some evidence that combined predator and harvest management of the Nelchina and Fortymile caribou herds altered natural cyclic tendencies (Boertje et al. 2017). We recommend that mathematical models be used to help determine the timing and amount of *Rangifer* harvest that may achieve cycle management via dynamical control theory. Our study shows how theoretical models and empirical research can help acquire both a better understanding of the drivers of population cycle gradients and help to put forth ecologically informed management strategies.

## Author Contributions

**T. J. Clark‐Wolf:** conceptualization (equal), data curation (equal), formal analysis (equal), writing – original draft (equal), writing – review and editing (equal). **Jack St. John:** data curation (equal), formal analysis (equal), writing – original draft (equal), writing – review and editing (equal). **Chandni A. Rajesh:** data curation (equal), formal analysis (equal), writing – original draft (equal), writing – review and editing (equal). **Mark Hebblewhite:** conceptualization (equal), supervision (equal), writing – original draft (equal), writing – review and editing (equal).

## Conflicts of Interest

The authors declare no conflicts of interest.

## Supporting information


Data S1.

## Data Availability

The code and data needed to reproduce this analysis can be found on Github and is archived on Zenodo: https://doi.org/10.5281/zenodo.15263624
