# Supplementary material for: Caribou and Reindeer Population Cycles Are Driven by Top‐Down and Bottom‐Up Mechanisms Across Space and Time
Source: Ecol Evol. 2025 May 7;15(5):e71348. doi: 10.1002/ece3.71348 (PMC12058453; doi:10.1002/ece3.71348)
Supplement: Supplementary file 1 — Data S1. [file ECE3-15-e71348-s001.docx]

**Caribou and reindeer population cycles are driven by top-down and bottom-up mechanisms across space and time**

Clark-Wolf *et al.*

**Supporting Information**

**Figure S1. Predicted *Rangifer* population time-series from our tri-trophic model.** Time-series period is equal to 57.7, and amplitude is equal to 0.947. See red dot in Fig.2.

**
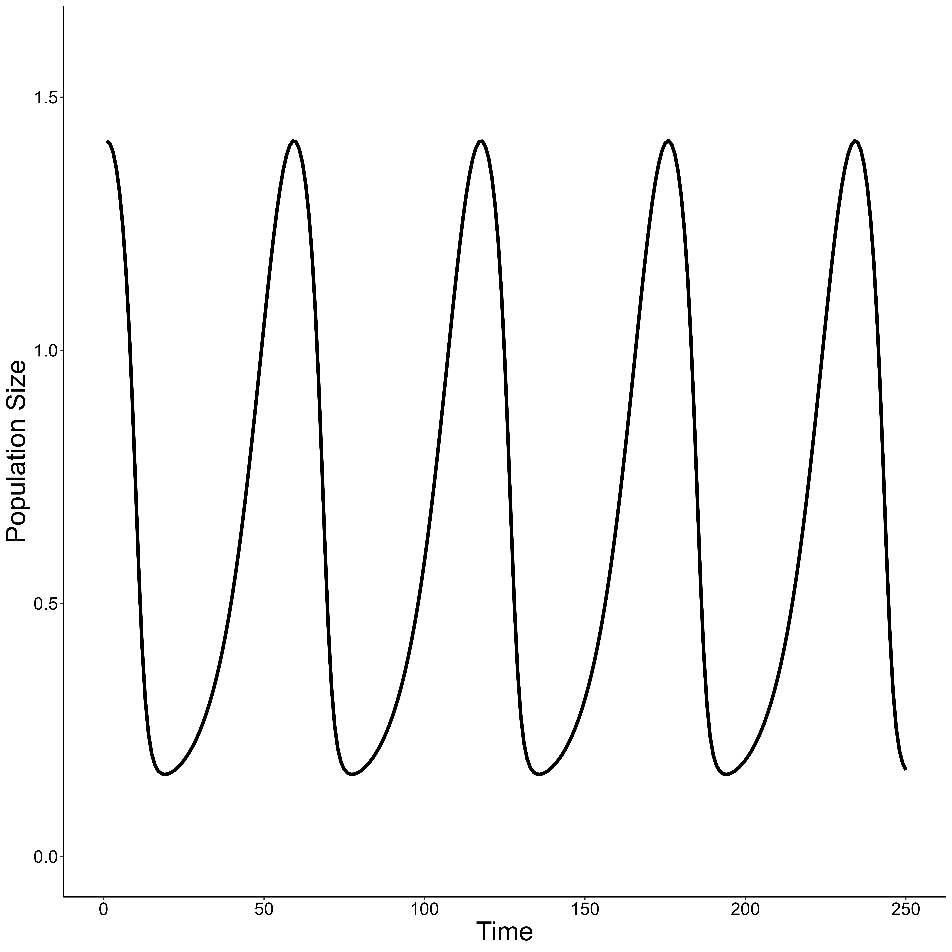
**

**Table S1.** *Rangifer* (caribou and reindeer) populations, total data length, and cycle period and amplitude if populations were found to be cyclic. “NA” in cycle period and amplitude indicates population was not found to be significantly cyclic.

| Population Name | Location | Data Length | Cycle Period | Cycle Amplitude |
| --- | --- | --- | --- | --- |
| Aasiaat | Greenland | 70 | NA | NA |
| Adak Island | USA | 55 | NA | NA |
| Adventdalen | Svalbard | 40 | NA | NA |
| Alakyla | Finland | 23 | 23 | 0.43 |
| Bathurst | Canada | 42 | 42 | 1.11 |
| Beverly | Canada | 52 | 52 | 0.49 |
| Bluenose East | Canada | 19 | NA | NA |
| Bluenose West | Canada | 33 | 33 | 0.83 |
| Broggerhalvoya | Svalbard | 41 | NA | NA |
| Cape Bathurst | Canada | 33 | 33 | 1.00 |
| Central Arctic | USA | 45 | 45 | 1.14 |
| Delta | USA | 39 | 39 | 0.99 |
| Denali | USA | 44 | NA | NA |
| Dolphin Union | Canada | 25 | NA | NA |
| Edgeoya | Svalbard | 38 | 38 | 0.71 |
| Fortymile | USA | 65 | 65 | 1.21 |
| George River | Canada | 67 | 67 | 1.57 |
| Haute Island | Kerguelen | 26 | NA | NA |
| Iceland | Iceland | 70 | NA | NA |
| Kainuu | Finland | 25 | 25 | 0.49 |
| Kaldoaivi | Finland | 23 | NA | NA |
| Kangerlussuaq | Greenland | 34 | 34 | 1.40 |
| Leaf River | Canada | 43 | 43 | 0.87 |
| Manitsoq | Greenland | 74 | NA | NA |
| Mentasta | USA | 31 | NA | NA |
| Muotkatunturi | Finland | 23 | NA | NA |
| Nelchina | USA | 66 | NA | NA |
| Nunivak Island | USA | 95 | NA | NA |
| Nuuk | Greenland | 74 | NA | NA |
| Palojarvi | Finland | 23 | NA | NA |
| Pammiut | Greenland | 73 | NA | NA |
| Porcupine | USA/Canada | 46 | 23 | 0.41 |
| Qamanirjuaq | Canada | 50 | 50 | 0.87 |
| Qeqer | Greenland | 24 | NA | NA |
| Reindalen | Svalbard | 40 | NA | NA |
| Sisimiut | Greenland | 74 | NA | NA |
| Southampton Island | Canada | 50 | 50 | 1.51 |
| St. Matthew Island | USA | 23 | NA | NA |
| Taimyr | Russia | 59 | 59 | 0.84 |
| Teshekpuk Lake | USA | 34 | NA | NA |
| Upernavik | Greenland | 46 | NA | NA |
| Western Arctic | USA | 50 | 50 | 0.70 |
| White Mountains | USA | 34 | 34 | 0.70 |

**Table S2.** Model selection results for *Rangifer* (caribou and reindeer) cycle period as a function of ecological variables. Models are ranked by ΔAIC.

| Model Structure | ΔAIC |
| --- | --- |
| Amp + Latitude + Subspecies + Biome + Temp + NDVI | 0 |
| Amp + Latitude + Wolves + Subspecies + Biome + Temp + NDVI | 1.93 |
| Amp + Latitude + Wolves + Predators + Subspecies + Biome + Temp + NDVI | 3.67 |
| Amp + Latitude + Wolves + Predators + Subspecies + Biome + Temp + NDVI + Precip | 5.66 |
| Intercept | 12.8 |

**Table S3.** Final model parameters for *Rangifer* (caribou and reindeer) cycle period as a function of ecological variables. Final model was chosen by ranking ΔAIC.

| Intercept | Coefficient | SE |
| --- | --- | --- |
| Intercept | 16.701 | 13.545 |
| Amp | 7.203 | 2.582 |
| Latitude | -9.481 | 5.894 |
| Subspecies - fennicus | 31.222 | 16.895 |
| Subspecies - granti | 21.612 | 11.133 |
| Subspecies - groenlandicus | 6.404 | 9.815 |
| Subspecies - platyrhynchus | 50.434 | 25.100 |
| Subspecies - tarandus | 30.568 | 16.881 |
| Biome – taiga/tundra | 16.777 | 7.273 |
| Biome - tundra/tundra | 4.296 | 8.490 |
| NDVI | -1.596 | 1.515 |
| Temp | -5.568 | 2.973 |

**Table S4.** Model selection results for *Rangifer* (caribou and reindeer) cycle amplitude as a function of ecological variables. Models are ranked by ΔAIC.

| Model Structure | ΔAIC |
| --- | --- |
| Per + Subspecies + Biome + Temp + NDVI | 0 |
| Per + Latitude + Subspecies + Biome + Temp + NDVI | 0.50 |
| Per + Latitude + Wolves + Subspecies + Biome + Temp + NDVI | 3.95 |
| Per + Latitude + Wolves + Subspecies + Biome + Temp + NDVI + Precip | 5.67 |
| Per + Latitude + Wolves + Predators + Subspecies + Biome + Temp + NDVI + Precip | 7.67 |
| Intercept | 8.50 |

**Table S5.** Final model parameters for *Rangifer* (caribou and reindeer) cycle amplitude as a function of ecological variables. Final model was chosen by ranking ΔAIC.

| Intercept | Coefficient | SE |
| --- | --- | --- |
| Intercept | 0.740 | 0.460 |
| Period | 0.064 | 0.021 |
| Subspecies - fennicus | -0.303 | 0.428 |
| Subspecies - granti | -0.213 | 0.206 |
| Subspecies - groenlandicus | 0.046 | 0.215 |
| Subspecies - platyrhynchus | -0.845 | 0.377 |
| Subspecies - tarandus | -0.272 | 0.252 |
| Biome – taiga/tundra | -0.232 | 0.265 |
| Biome – tundra/tundra | 0.209 | 0.236 |
| NDVI | -0.094 | 0.037 |
| Temp | -0.007 | 0.013 |

**Table S6.** Sensitivity analysis of cycle period in tri-trophic model. Values show cycle period in response to changes in parameter values.

| Change in Parameter | u_0_ | m | a | b | ξ | η | c | d | X | μ | s_0_ | κ |
| --- | --- | --- | --- | --- | --- | --- | --- | --- | --- | --- | --- | --- |
| +30% | 57.7 | 60.0 | 44.1 | 65.2 | 51.7 | 57.7 | 71.4 | 45.5 | 57.7 | 26.8 | 57.7 | 60.0 |
| +15% | 57.7 | 57.7 | 50.0 | 62.5 | 55.5 | 60.0 | 65.2 | 51.7 | 57.7 | 45.5 | 57.7 | 57.7 |
| 0% | 57.7 | 57.7 | 57.7 | 57.7 | 57.7 | 57.7 | 57.7 | 57.7 | 57.7 | 57.7 | 57.7 | 57.7 |
| -15% | 57.7 | 57.7 | 71.4 | 53.6 | 62.5 | 55.5 | 55.5 | 62.5 | 57.7 | 65.2 | 57.7 | 57.7 |
| -30% | 57.7 | 55.5 | 93.7 | 48.4 | 68.2 | 53.6 | 47.7 | 65.2 | 60.0 | 75.0 | 60.0 | 57.7 |

**Table S7.** Sensitivity analysis of cycle amplitude in tri-trophic model. Values show cycle amplitude in response to changes in parameter values.

| Change in Parameter | u_0_ | m | a | b | ξ | η | c | d | X | μ | s_0_ | κ |
| --- | --- | --- | --- | --- | --- | --- | --- | --- | --- | --- | --- | --- |
| +30% | 1.20 | 0.99 | 0.95 | 0.88 | 0.98 | 0.52 | 1.61 | 0.28 | 0.94 | 0.11 | 0.93 | 0.94 |
| +15% | 1.09 | 0.98 | 0.96 | 0.92 | 0.96 | 0.68 | 1.30 | 0.69 | 0.95 | 0.45 | 0.94 | 0.94 |
| 0% | 0.95 | 0.95 | 0.95 | 0.95 | 0.95 | 0.95 | 0.95 | 0.95 | 0.95 | 0.95 | 0.95 | 0.95 |
| -15% | 0.70 | 0.89 | 0.89 | 0.96 | 0.92 | 1.10 | 0.65 | 1.14 | 0.94 | 1.33 | 0.93 | 0.93 |
| -30% | 0.54 | 0.79 | 0.75 | 0.98 | 0.89 | 1.24 | 0.33 | 1.47 | 0.93 | 1.82 | 0.95 | 0.92 |
